# The frog hand illusion: Distortion of hand shape in inverted presentation

**DOI:** 10.1177/20416695251319270

**Published:** 2025-02-27

**Authors:** Shuichiro Taya, Achille Pasqualotto

**Affiliations:** 26322 Hiyoshi Psychology Laboratory, Keio University, Japan; 13121 Institute of Human Sciences, University of Tsukuba, Japan

**Keywords:** visual illusion, perceptual constancy, space perception, perceptual inference, multisensory interaction, frog hand illusion

## Abstract

When a photograph of the back of a hand with the fingers extended to the depth is observed upside-down, the hand appears vertically squashed, with extremely short fingers. The first aim of this study was to quantitatively measure the “frog hand illusion (FHI)”, named after its bizarre appearance, and the second aim was to examine whether the dominant hand affects the strength of FHI. We measured the apparent shortening of the fingers using the method of constant stimuli. The results showed that the fingers of the inverted hand appeared to be shorter than those of the upright hand by about 5% on average. No effect of the dominant hand was observed. We propose the hypothesis that FHI occurs because of the attenuation of perceptual constancy, which might stem from observing the hand image from an atypical viewpoint.

## How to cite this article

Taya, S., & Pasqualotto, A. (2025). The frog hand illusion: Distortion of hand shape in inverted presentation. *i–Perception*, *16*(0), 1–9. https://doi.org/10.1177/20416695251319270

## Introduction

The sensory input from retinal images is inherently ambiguous and open to multiple interpretations due to internal and external noise. Therefore, the visual system is constantly required to perform inferences by narrowing down the possible answer from countless options. In the process of this inference, the visual system is guided by frameworks based on the prior knowledge (e.g., Bayesian inference; Ma et al., 2023), with the sense of body posture being one prominent prior (Romano et al., 2019). Bodily sensations can help constrain visual interpretation because our visual experiences are consistently associated with specific bodily positions and the sense of gravity. For instance, the coordinates of retinal images are often represented in conjunction with bodily sensory and gravitational axes (Azañón et al., 2016; Higashiyama & Adachi, 2006). Our visual perception is trained through repeated presentations where retinal images are coupled with bodily sensations that contribute to achieving perceptual constancy; the perceived world is stable despite substantial changes in retinal images due to variations in viewing conditions (viewing distance, viewing angle, lighting, etc.). For this reason, deviations from the typical coupling of body posture and retinal images, such as the inversion of retinal images, result in alterations in visual perception, including visual illusion (e.g., Rock, 1974, 1988).

An example of how atypical observation conditions can cause changes in our perception, especially in terms of perceptual constancy, is demonstrated by the phenomenon known as the “between-legs effect” (Helmholtz, 1866/Higashiyama & Adachi, 2006). When a landscape is observed by looking upside down between the legs, size constancy is deteriorated and the farther away the object in the scene is, the smaller it is perceived: i.e., the apparent size of an object becomes highly dependent on the size of its retinal image. This was already pointed out by Helmholtz in the 19th century, who attributed this phenomenon to the atypicality of the retinal image; he explained that the upside-down visual field itself distorts the function of size constancy. According to Higashiyama and Adachi (2006), however, the effect is due to somatosensory atypicality. In their experiment, when the subjects did not observe a landscape between their legs (i.e., their somatosensory perception was typical) and simply inverted the visual field with prism glasses (i.e., the retinal image was atypical), the size perception was not different from that in the upright viewing condition. In contrast, when the scene was observed between the observers' legs with their head down (i.e., somatosensory perception was atypical) and inverted the visual field with a prism (i.e., the retinal image was upright = typical), they found the reduction in constancy as seen in the between-legs effect. In short, Higashiyama and Adachi's results suggest that it is the atypicality of somatosensory perception, and not the mere inversion of the retinal image that causes the attenuation of size constancy.

**Figure 1.**
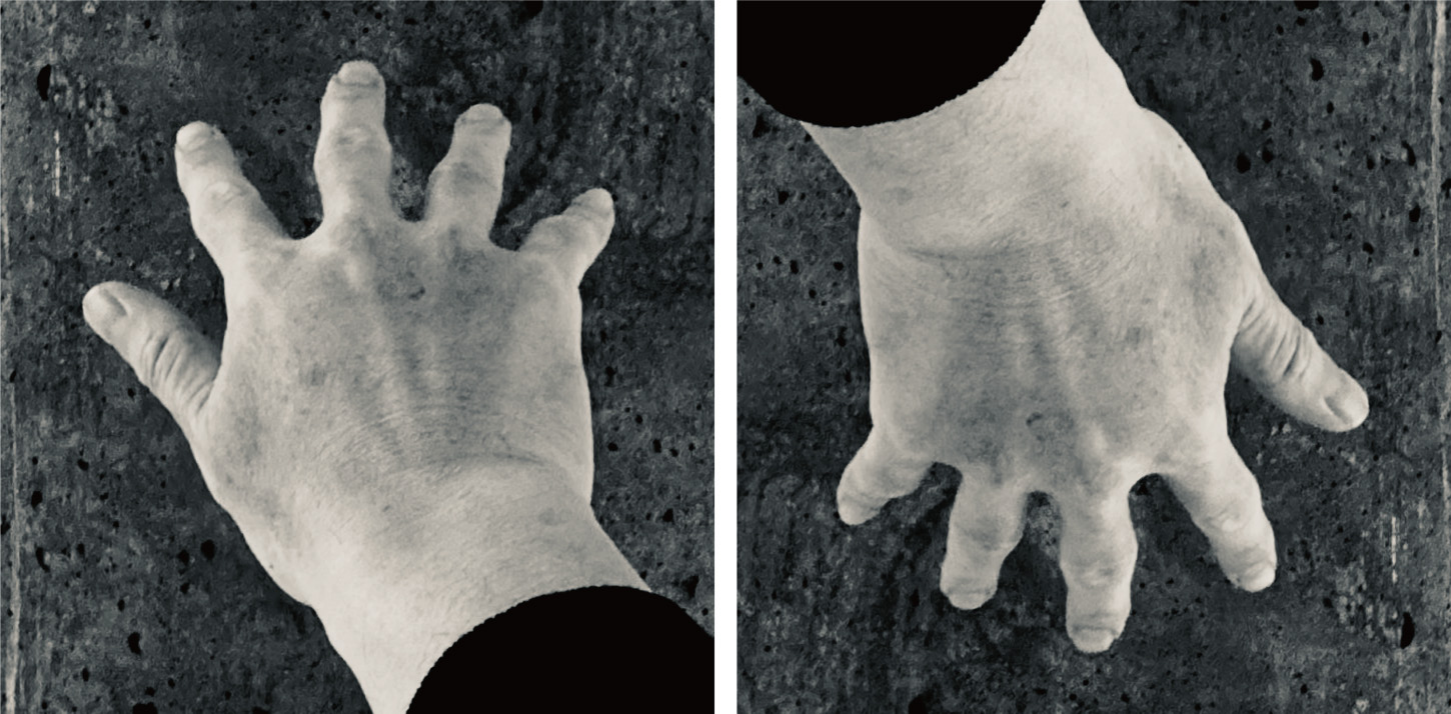
The frog hand illusion. The right image is simply the left one turned upside down, but a distorted hand with extremely short fingers is perceived.

In contrast, we here report a phenomenon suggesting that, when we see a body image, even if the observer's body posture is typical, that is, upright, size constancy can be weakened simply by inverting the retinal image. In the right image of Figure 1, many observers perceive an oddly shaped hand with strangely short fingers; however, the right image is simply an upside-down version of the left image. Due to this apparent distortion, we refer to this phenomenon as the “frog hand illusion (FHI).”

Why do the fingers appear shorter when viewed simply upside down? To consider the reason, we should note that, in Figure 1, the fingers are depicted extremely short regardless of whether the image is upright or inverted. This foreshortening occurs purely due to optical effects; the fingers appear shorter in the photograph because they are positioned obliquely rather than perpendicularly to the line of sight. Naturally, this foreshortening is independent of the image's orientation. However, the crucial point is that when we view the upright image, the fingers do not appear short. This is likely due to the function of size/shape constancy. Thus, FHI can be understood as a phenomenon arising from the weakening of the constancy in the inverted image (see the “Discussion” section for more details).

In the following sections, this article presents the results of psychophysical measurement of the FHI. We then propose and discuss the hypothesis that the weakening of constancy, which we posit as the cause of this illusion, might be due to the atypicality of the viewpoint of the presented body image.

## Methods

The experiment was designed with two objectives. One was to quantitatively demonstrate the illusion magnitude. The other was to examine the influence of handedness on the FHI.

For these purposes, 24 right-handed observers (12 males and 12 females) recruited among the students at the University of Tsukuba participated in the experiment (mean age = 22.38, SD = 2.92). They had normal or corrected-to-normal vision and signed the Informed Consent Form approved by the Research Ethics Committee at the Faculty of Human Sciences. They received a 500-yen voucher for their participation.

**Figure 2.**
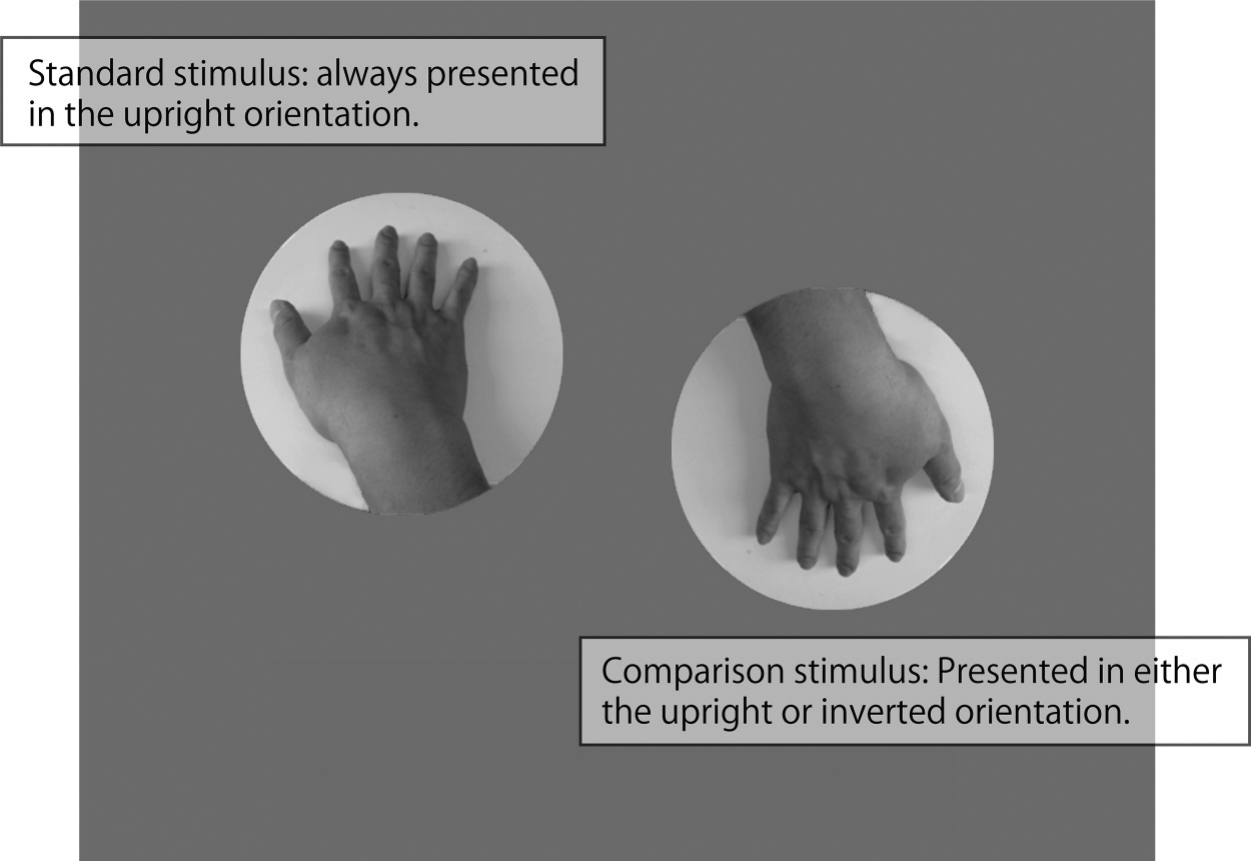
An example of the stimulus display (from the inverted condition).

The illusion magnitude was measured using the method of constant stimuli. The standard stimulus was always presented upright, while the comparison stimulus was displayed either upright or inverted. Henceforth, the condition with the upright comparison stimulus will be referred to as upright condition, and the condition with the inverted comparison stimulus as inverted condition. On a PC screen, a standard stimulus without alteration to the aspect ratio was presented alongside a comparison stimulus that was either vertically compressed or stretched. The task of the participants was to indicate, in a two-alternative forced choice (2AFC) format, which of the two-hand images appeared to have longer fingers. Greyscale images of a right and a left hand were used as the stimuli. All stimuli were from the same original, photographed from the same individual. The comparison stimuli were photographs in which the aspect ratio of these original photographs was vertically stretched (or shrunk) so that it became (height/width) 0.88, 0.92, 0.96, 1.00 (i.e., the original), 1.04, 1.08, and 1.12 of the original. Additionally, all these upright images were rotated by 180° to create inverted images (see Figure 2). All photographs were cropped and presented within a 300-pixel diameter circle. This manipulation meant that nothing other than the hand and the wrist were reflected in the stimulus presentation area except the shadow of the hand and the supporting surface with no background (Figure 2).

After signing the consent form, participants filled out the questionnaires from the English or Japanese version (depending on the origin of the participants) of the Flanders Handedness Test (Nicholls et al., 2013; Okubo et al., 2014). Participants answered “left,” “right,” or “ambidextrous” to a 10-item questionnaire printed on an A4 sheet. Then, they sat about 60 cm from the computer screen with their heads on a chinrest.

Stimulus presentation and data collection were automated using PsychoPy3 (Version 2022.2.4; Peirce et al., 2019). Instructional text was presented in Japanese and English on the screen at the beginning of the experiment. During each trial, a reminder text “Choose the hand you think has longer fingers by using the arrow keys (<=/=>)” was presented, and the standard and comparison stimuli were presented side by side for 1500 ms.

The images disappeared after 1500 ms, but the instructions remined until the participant responded with a key press. No time limit was set for the response. The next trial started immediately after the key press response. In half the trials, the standard stimulus appeared on the right side and the comparison stimulus on the left side of the screen (and vice versa). The sides of the comparison and standard stimuli were randomly determined. The experiment included two sessions, one using right hands and one using left hands, counterbalanced across participants. Each session had 10 blocks of 14 trials (thus, 280 trials in the entire experiment). Participants could take a short rest between blocks. In one block, all seven comparison stimuli, either upright or inverted, were presented once in a random order and matched with the standard stimuli. Psychophysical functions were obtained by fitting with 10 data points (one per block) for each of the eight comparison stimuli. The experiment lasted about 30 minutes.

## Results

For each of the four experimental conditions (right- and left-hand ×upright and inverted), a cumulative normal distribution function was fitted to the responses of each of the 24 participants, and the 50% point was determined as the point of subjective equivalence (i.e., PSE, the length of the comparison stimulus that was deemed to have appeared equal to the length of the standard stimulus). In the inverted presentation condition, a total of eight participants were unable to obtain the PSE because they always judged the inverted comparison stimulus to have appeared “longer.” This issue occurred in both the left-hand condition and the right-hand condition, with four participants affected in each condition. Data from these eight participants were excluded from subsequent analyses.

**Figure 3.**
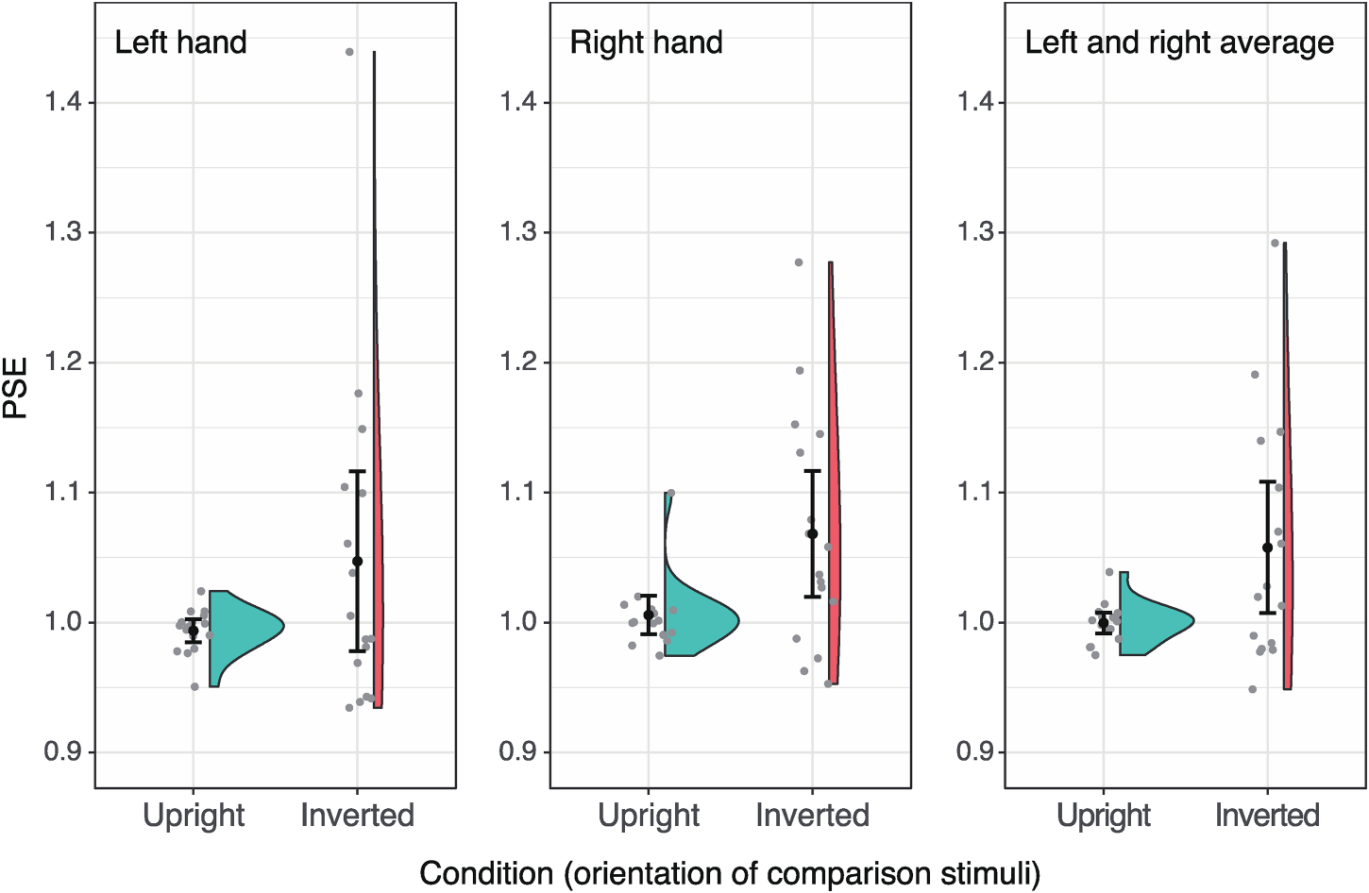
The results of the experiment for left-hand stimuli, right-hand stimuli, and their average (from left to right). The PSE greater than 1 indicates that the comparison stimulus needed to be enlarged to match the standard stimulus (that was always upright), meaning that the comparison stimulus appeared shorter than the standard stimulus. The black circles with error bars represent group PSE means with 95% confidence intervals, while light grey circles indicate PSE for each observer.

The results of the experiment quantitatively corroborated the appearance of the FHI. Figure 3 shows the measurement results (individual PSEs and group averages for 16 participants) for left-hand stimuli and right-hand stimuli, and their average. It is clear from the figure that the group mean of the PSEs for the inverted presented hand was 4% to 6% longer than the upright image, that is, the fingers in the inverted image were perceived as shorter than in the upright image. A within-subjects two-factor analysis of variance (left/right × upright/inverted) using 16 PSEs showed that only the main effect of stimulus orientation (upright/inverted) was significant (*F*_1,15_ = 5.93, *p* = 0.03, *η*^2^ = 0.12) (see Figure 3). The main effect of the left/right hand and the interaction between the factors were not significant (*F*_1,15_ = 0.96, *p* = 0.34, *η*^2^ = 0.06; *F*_1,15_ = 0.10, *p* = 0.75, *η*^2^ = 0.00, respectively).

Did the inversion of the stimulus image also affect the accuracy of responses? To examine this point, we also analysed the standard deviation of individual responses: that is, the slope of the psychometric function. For this analysis, we used data from the same 16 participants as in the analysis of the PSEs. However, no significant main effects were found for image orientation (upright or inverted), hand image (left or right), or their interactions (*F*_1,15_ = 1.04, *p* = 0.33, *η*^2^ = 0.02; *F*_1,15_ = 0.97, *p* = 0.34, *η*^2^ = 0.02; *F*_1,15_ = 0.17, *p* = 0.68, *η*^2^ = 0.00, respectively). Therefore, while the inversion of the image altered the apparent length, it did not significantly increase variability in judgment.

The homogeneity in sample variance between the upright and inverted conditions was also examined. A Pitman-Morgan test on the variance of the left-right average data (Figure 3, right) showed significantly greater variance in the inverted condition (*t*_14_ = 11.42, *p* = 1.77 × 10^−8^). Yet, as the data fitting had no issues, this difference likely reflects individual variability in the illusion magnitude rather than measurement artifacts. If the FHI arises from perceptual inference of 3D structures, this variability may be linked to individual differences in the inference process. For instance, individual variations in priors used for recovering 3D space based on pictorial cues (e.g., Knill, 2007; Taya & Sato, 2018) could explain this difference.

## Discussion

The primary aim of this study was to quantitatively measure the FHI. The results supported qualitative observations, showing that in the inverted hand images, fingers appeared shorter than in upright images. The secondary aim was to examine whether handedness influenced the FHI's strength. However, no effect of handedness was observed; all participants were right-handed and experienced a similar degree of illusion with both left and right hand images.

As with the FHI, illusions observed in unprocessed photographs have been reported. For instance, when two identical photographs of the Tower of Pisa showing the tower tilted to the right are placed side by side, the right one appears to tilt further (Kingdom et al., 2007). Similarly, in a photograph of a straight road, the angle between the centre line and the sideline of the road, which is more than 90° (obtuse angle), looks like to be an acute angle (Osa et al., 2011). These illusions are primarily explained by mechanisms of size and shape constancy that reconstruct three-dimensional structures from two-dimensional retinal images. The visual system compensates for changes in retinal images caused by differences in viewing distance and perspective, allowing us to perceive the true size and shape of objects. We believe that FHI arises from the same mechanism. As previously mentioned, in a photograph of a hand with fingers extended into depth, the fingers appear foreshortened due to perspective projection. Normally, size/shape constancy compensates for this, but the mechanism fails with an inverted image of the hand, resulting in the FHI. In other words, the optical foreshortening of the fingers is interpreted as an actual shortness of fingers in real space.

The diminishment of constancy in an inverted photo can be understood under the view that constancy is the result of perceptual inference based on our prior knowledge; since our perceptual inference depends on past experiences, this process becomes distorted if we have little experience about them, like an inverted image of a scene. Empirical evidence supporting this idea comes from the studies reporting that some illusions, which are considered to be caused by size constancy, are diminished with upside-down image presentation (Coren, 1992; Fujita, 1996; Osa et al., 2011). However, those illusions in the previous studies appeared in photographs or drawings of a landscape, and unlike a landscape, a hand has a greater degree of freedom in terms of perceived orientation; shortly speaking, it is unclear which is the top and which is the bottom. Thus, it is challenging to simply apply this explanation directly to the FHI.

We propose that the atypicality of the viewpoint, which induces the FHI, might come from the biomechanical constraints of our body. That is, the FHI image represents an atypical viewpoint rarely encountered in daily life, resulting in a limitation of the constancy mechanism and thus producing the illusion. Readers might wonder why the pose of pointing fingers downward and in-depth is considered atypical. However, it is essential to note that the FHI-triggering image shows the back of the hand. A moment's reflection will reveal that if the hand in view is the observer's own, it is indeed rare to see one's fingers pointing downward and in-depth with the back of the hand visible. Typically, when the back of the hand points in-depth, the fingers are oriented upward. For the back of the hand to appear as it does on the right side of Figure 1, an unusual or at least awkward posture is required, one not often assumed in daily life. For example, placing the hand beside the body, twisting the wrist to turn the back of the hand toward the torso, and looking down at it might result in a view like the one in Figure 1 right. However, it is rare to assume such a posture.

Some may still argue that if the back of the hand belongs to someone else, seeing a hand with fingers pointing downward, as in Figure 1 right, is not particularly uncommon. Nevertheless, we argue that the fingers pointing in-depth decreases its typicality. For example, a typical posture for viewing the back of someone else's hand with their fingers pointing downward would be when the person has his/her arm extended straight down from the shoulder. However, to obtain a retinal image of the back of his/her hand with the fingers pointing away in-depth, you would need to stand close to the person, bring your face near his/her shoulder, and look down. This is not considered a typical viewpoint. Furthermore, there is empirical evidence suggesting that we implicitly assume typical body positioning, which also supports the interpretation that an image like the one in Figure 1 right is considered as atypical (Romano et al., 2017, 2019). These studies reported that the thumb is generally assumed to be positioned below the other fingers, which is opposed to the finger position in the FHI image (Figure 1, right).

In addition, it cannot be ruled out at this point that the FHI may be a phenomenon caused by visual stimuli eliciting somatosensory signals. It has been reported that when observing a human body or its parts, the range of motion of body joints influences visual perception even if observers do not move their own bodies. For example, in a mental rotation task in which line drawings of the right and left hands are rotated at various angles as stimuli and the observer is asked to judge whether the image depicts the right or left hand, the relationship between the rotation angle of the image and reaction time has been shown to be asymmetric, depending on the range of motion of the joints of the human body (Parsons, 1987; Sekiyama, 1982). Similarly, in a task in which participants were asked to judge whether two images of a body assuming different poses were the same or different, it was shown that reaction times were shorter when stimuli were presented in poses that could actually be taken by humans than when stimuli were presented in poses that could not be taken, given the range of motion of the joints (Inoue & Kitazaki, 2010; Reed et al., 2003). These studies suggest that when viewing images of a body or its parts, the visual system automatically takes biomechanical constraints into account. In the case of FHI, when viewing an image of a hand, the visual system might adopt the most plausible interpretation of the posture that the owner of the hand is likely to have taken under biomechanical constraints. As explained above, in the case of the back of a hand with the fingers pointing downward, it is unlikely that the fingers are pointing in-depth. Consequently, the visual system does not interpret the foreshortening as an effect of optical projection, leading to the perception of shorter fingers (FHI). As mentioned in the introduction, Higashiyama and Adachi (2006) reported that what weakens perceptual constancy is not the atypicality of the retinal image itself but the atypicality of somatosensory signals. At first glance, their claim appears to contradict our findings, which suggest that the mere inversion of the retinal image weakens constancy. However, this apparent contradiction can be resolved if we consider that the atypicality of the visual image of a hand mediates the atypicality of somatosensory signals in the FHI.

In conclusion, we suggest that the weakening of depth perception due to the atypicality of viewpoint is a key factor in the FHI. From this perspective, whether the FHI is a phenomenon specific to the body remains open to debate; that is, similar illusions may occur with non-body objects if constancy is weakened by an atypical viewpoint. For example, objects frequently used by hands, such as scissors, may develop similar typicality of viewpoint (i.e., prior), potentially resulting in effects similar to the FHI. Put differently, body images might be a sufficient condition but not a necessary condition for producing the FHI. However, since objects generally have fewer constraints on viewing directions than the body, and there are few objects viewed as frequently as the body, the magnitude of the illusion for objects may be smaller or negligible (our informal observations support this idea). In this respect, the fact that the images are of a body might hold some significance. Regardless, further investigation is needed to address this issue.
